# Study of Operational Parameters on Indium Electrowinning Using a Ti Cathode

**DOI:** 10.3390/ma18092089

**Published:** 2025-05-02

**Authors:** Carla Lupi, Erwin Ciro, Alessandro Dell’Era

**Affiliations:** 1 Department ICMA, Sapienza University of Rome, Via Eudossiana 18, 00184 Roma, Italy; carla.lupi@uniroma1.it (C.L.); e.ciro@lab.unimarconi.it (E.C.); 2 Department SBAI, Sapienza University of Rome, Via del Castro Laurenziano 7, 00161 Roma, Italy

**Keywords:** indium recovery, electrowinning, sulfate solution, titanium cathode

## Abstract

Indium, widely used as indium-tin oxide (ITO), has been recognized as a strategical metal for audiovisual, optoelectronic systems, semiconductors and photovoltaic fields. An increasing shortage and unflexible mineral supply have led indium to be recovered from secondary sources, such as waste electrical and electronic equipment (WEEE). The main step for indium hydrometallurgical recovery from WEEE is the electrowinning process using sulfate baths, giving lower environmental impact and improved workplace safety conditions. In this investigation, a titanium cathode has been employed for the study of the indium electrowinning process in a sulfate-based bath. This study was focused on analyzing current efficiency (CE), specific energy consumption (SEC) and deposit morphology and structure as the temperature, current density, pH and electrolyte composition were varied. Prior to conducting electrowinning tests, a conventional three-electrode cell was used to perform cyclic voltametric assessments of the electrodeposition reactions on the Ti electrode at room temperature. The indium electrowinning tests on Ti cathodes presented CE values higher than 90%, with low energy consumption at low current densities, showing a negligible influence of additive agents in the bath, different from results obtained with other cathodes in other works. Moreover, the increase of the current density beyond 75 A/m^2^ produced significant effects by etching the electrode surface with 1M HF. In particular, at the conclusion of this investigation, good results are obtained without additives, by etching the titanium cathode and operating at higher current density between 100 and 200 A/m^2^ at pH 2.3 and different temperatures (40 °C and 60 °C). Finally, indium deposits were analyzed by XRD and SEM in order to determine the influence of operative conditions on the structure and surface morphology.

## 1. Introduction

Indium, recognized as a strategic metal, has gained significant importance across several technological fields, particularly in the electronics and semiconductor industries, as well as in coatings, nuclear reactors, sensors and biomedical applications [1,2,3,4,5]. Among semiconductor devices, indium is usually utilized in the form of indium-tin oxide (ITO) because of its high conductivity and transparency, making it fundamental for touch panel devices, liquid-crystal displays (LCDs), organic light-emitting diodes and photovoltaic cells [6,7]. In recent years, the exceptional properties of ITO have driven a surge in consumption, leading to rising global demand and causing an imbalance in the indium market. This critical scenario has been worsened by limited mineral resources, geopolitical restrictions and the accelerated production of high-tech devices [8,9]. It is well established that indium extraction from primary ore deposits is challenging, as indium can only be found in small quantities within aggregates or as a by-product of ores such as sphalerite, chalcopyrite and galena [10,11,12]. With restricted controls over the international indium supply, a critical shortage is increasingly likely in the near future. Consequently, the search for either alternative extractable ores or novel sources of indium has become one of the most important strategies to mitigate its forthcoming scarcity and ensure the continued development of emerging technological fields.

Regarding indium extraction, its limited availability in the Earth’s crust makes the discovery of novel primary ores increasingly unlikely, rendering the beneficiation of indium-bearing minerals improbable or even economically unfeasible. Nowadays, secondary sources, such as waste electrical and electronic equipment (WEEE), have emerged as unexploited raw materials with considerable benefits. These include reducing occupational risks associated with mining due to the absence of radioactive metals (Th and U), decreasing power and reagent consumption during extraction and providing higher metal concentrations for beneficiation [13,14,15,16,17]. Thus, indium recovery from WEEE could represent a promising alternative to supply and complete with the growing annual demand for this critical metal. Indium recovery from WEEE has been carried out by both pyrometallurgical and hydrometallurgical methods. In pyrometallurgical processes, pyrolysis and vaporization are widely employed to recover indium at high temperatures, commonly exceeding 1100 °C [18,19,20,21,22,23]. Nevertheless, several critical environmental and economic drawbacks should be considered before industrial implementation, including high cost for facilities and equipment, substantial energy consumption and the generation of hazardous, and complex by-products. On the other hand, hydrometallurgy is considered the most versatile strategy for recovering indium from ITO in LCD scraps [24,25,26,27,28,29,30,31,32,33]. The process involves leaching ITO using various aqueous solutions containing nitric, hydrochloric or sulfuric acid, followed by concentration and purification treatments [33,34,35,36] and electrowinning to recover pure indium metal. Although efforts to develop pilot scale projects or plants proposals for indium recovery have not been promoted so far, research on optimization of the operative parameters has increased significantly in recent years. The main electrowinning operative parameters include current density (CD), bath composition, temperature, pH and the type of electrodes or reactors used.

Several investigations have been conducted into nitrate baths at a CD of 64 A/m^2^ using Al/Fe cathodes at room temperature to recover indium, aiming for CE values exceeding 90% with a low SEC of 0.5 kWh/kg [37]. Other studies have focused on chloride baths in order to evaluate the effects of the additive agents such as LiCl and NaCl, with copper as the cathode [38]. Similarly, Lee and Oh conducted experiments using an electrolyte containing InCl_3_-HCl-NaOH and additive agents (NaCl and InCl_3_) at a CD of 0.100 A/m^2^, achieving high-purity indium with high CE [39]. In general, the indium concentration in electrolytes used for electrowinning with chloride-based acidic baths ranges from 0.13 to 26 g/L, producing deposits with a variety of morphologies and grain sizes (50–200 μm). Additionally, recent studies have explored electrolytes composed of NH_4_Cl for extracting oxide metals of In, Ga, and Zn present in ITO scraps [32].

Most electrowinning processes utilizing chloride and nitric acidic baths achieve CE values above 80%, regardless of the CD, maintaining low SEC and high-purity deposits. However, both electrolyte types present critical environmental, occupational safety and economic challenges. Other types of electrolytes, such as sulfate-based solutions, offer an alternative distant from plant costs. Although sulfate-based baths generally show lower efficiencies rates, these solutions exhibit reduced corrosion effects, lower costs and safer processing conditions compared to those electrolyte types mentioned above, thereby promoting the further development of indium electrowinning. Pioneering indium electrodeposition and recent studies examined the influence of operative parameters on both current efficiency and deposit morphology [40,41,42,43,44,45,46,47,48,49]. However, despite the notable advantages of sulfate-based baths, research on indium electrorefining and electrowinning remained scarce over the subsequent 40 years. In recent years, the importance of indium recovery processes has grown due to its strategic role in global metal markets. For instance, Xu et al. in 2021 proposed an extraction methodology of indium, in which electrorefining of crude indium was conducted to obtain high-purity indium deprived of Cd, Pd and Sn [50]. Similarly, Illés, I. B. et al. in 2022 developed a combined electrometallurgical approach, in which metals from LCD scraps were cemented and cast, followed by an electrorefining to produce indium with 99.9% purity [51].

Moreover, considering the interaction between indium and metal cathodes, other works have investigated the mechanism of electrodeposition reaction kinetics using cyclic voltammetry and chronoamperometry techniques, with Ti, Al and Cu as cathodic surfaces [52,53]. Several electrowinning processes have been studied using various metal cathodes, including AISI 316L, Ni, Ti, Al and Cu, while maintaining bath composition, temperature, pH, and CD at 140 g/L In_2_(SO_4_)_3_, 5 g/L H_3_BO_3_, 30 g/L Na_2_SO_4_ and 20 g/L Al_2_(SO_4_)_3_ at 40 °C, pH of 2.3 and 25 A/m^2^, respectively [54]. In particular, AISI 316L and Ni showed the highest productivity, achieving the highest CE and lowest SEC at approximately 93% and 1.7 kWh/kg, respectively [13,45,54,55].

Since the operative parameters (temperature, bath composition, pH and CD) in that research were kept unchanged, further variations in these parameters for each specific cathode could produce valuable insights into optimizing productivity. In this context, AISI 316L and Ni cathodes were investigated to assess the effect of operative parameters on the effectiveness of indium electrowinning [13,45]. In particular, using AISI 316L, the process recovery achieved around 80% CE and 2.7 kWh/kg under 25 A/m^2^, pH of 2.3, temperature of 40 °C and an electrolyte containing 60 g/L In^3+^, 20 g/L H_3_BO_3_, 30 g/L Na_2_SO_4_ and 20 g/L Al_2_(SO_4_)_3_. Meanwhile, Ni cathode yielded two notable results at two different CDs: the indium extraction performed at 80 A/m^2^ showed 98% CE and 1.7 kWh/kg SEC, whereas at 100 A/m^2^, 83% CE and 2.4 kWh/kg SEC were obtained. These findings indicate that the effectiveness of indium electrowinning is strongly influenced by the additives and cathodic material, as observed in the comparative investigation between AISI 316L and Ni cathodes at the same pH and temperature conditions [55]. Therefore, the present investigation, utilizing titanium cathode, focuses on analyzing the influence of the operative parameters on the indium electrowinning process in terms of CE, SEC and deposit morphology and structure. The results demonstrated a notable improvement in productivity and energy consumption compared to previously studied cathodes. Consequently, the findings suggest that employing sulfate baths and Ti cathodes provides a suitable methodology for efficient indium recovery with a lower environmental impact.

## 2. Materials and Methods

In the present study, the electrolyte has been prepared by using distilled water with the addition of anhydrous In_2_(SO_4_)_3_ (≥99.9%wt, Sigma Aldrich, St. Louis, MO, USA), H_3_BO_3_ (≥99.5%wt., Carlo Erba, Milan, Italy) and Na_2_SO_4_ (≥99.5%wt., Carlo Erba, Milan, Italy). In particular, the electrolyte was obtained by dissolving 70 g/L In^3+^ in distilled water, while changing the concentration of H_3_BO_3_ [56], and Na_2_SO_4_ with constant stirring. The pH varied from 1.5 and 2.7 values adding either caustic soda or sulfuric acid to determine its effect. Firstly, cyclic voltammetry tests were used to evaluate indium electrodeposition reaction on Ti in the sulfate solution. Cyclic voltammetry tests were carried out at room temperature with a scan rate of 10 mV/s and 20 mV/s. A three-electrode cell was used to evaluate the electrochemical behavior on Ti cathode in a potential range between −1.8 and 1.0 V vs. SCE. In this arrangement, four-grade titanium was cut into square samples of around 4.3 cm^2^ to be used as the working electrode embedded in polymethyl-methacrylate to expose only one cathodic face to the electrolyte, while a 99.99% platinum sheet was used as counter electrode and the reference electrode consisted of a KCl saturated Calomel glass body electrode (SCE, ESHE = +241 mV). Furthermore, cathodic surfaces were treated by polishing with silicon carbide sandpapers and then washing using distilled water. This cleaning also included the counter electrode through immersions into diluted nitric acid solutions followed by abundant distilled water washes. Taking into account the galvanostatic tests, a Pb-0.7Ag alloy plate, as the anode, was used and put 30 mm apart from the cathode and with a surface of approximately 6 cm^2^. The anodic surface was slightly higher than the cathodic surface, as larger anodic surfaces hinder unwanted dendritic structures caused by the edge effect on the cathodic boundaries. Moreover, both the titanium cathode and Pb anode, before and after each test, were pretreated by 1000-grit sandpaper to remove residual metals and oxides from the surface, which could increase electrode overpotential. A potentiostat/galvanostat (Amel Instruments 2053, Milan, Italy) was used to perform the cyclic voltammetry tests and the galvanostatic assessments with an accuracy ±0.2%. The CE values were obtained by weighting dried indium deposits and calculating the relationship between the real weight and the theoretical one. The real weight was obtained by the analytical balance (Mettler Toledo MX205DU, Columbus, OH, USA) with five digits of precision. Then the SEC can be calculated by knowing the CE and cell voltage (CV). Galvanostatic tests were repeated twice if the distance value between them was lower than 2%, otherwise the test was again repeated. In the graphs the best results were reported.

The morphology of indium deposits obtained on Ti electrode were analyzed by scanning electron microscope equipped with energy dispersive X-ray analyzer (Hitachi S-2500, SEM/EDS, Tokyo, Japan). Furthermore, the crystallographic assessments were carried out using a X-ray diffractometer (XRD) (PHILIPS PW 1830, Amsterdam, The Netherlands) with a Cu Kα source: λ = 1.5418 Å. A usual 2θ-slow scanning angle between 30° and 75° was used and crystallography open database (C.O.D) was used to identify the crystallographic patterns in samples.

## 3. Results and Discussion

### 3.1. Cyclic Voltammetry

**Figure 1:**
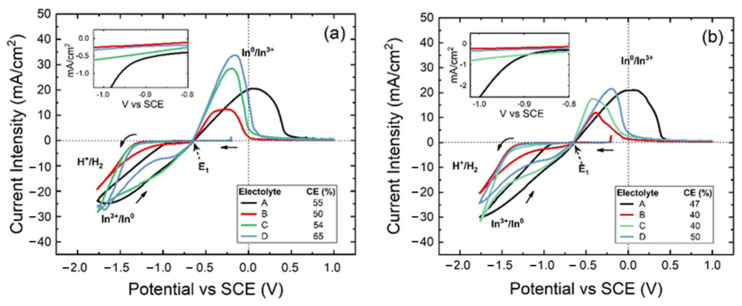
Cyclic voltammetry performed at (**a**) 10 mV/s and (**b**) 20 mV/s using a composition electrolyte of indium sulfate (A, black line); indium sulfate and boric acid (B, red line), indium and sodium sulfate (C, green line) and indium sulfate with both boric acid and sodium sulfate (D, blue line).

Cyclic voltammetry tests have been carried out at scan rate values of 10 mV/s and 20 mV/s in order to analyze the behavior of the reduction reactions and the CE. Different compositions of electrolytic solutions were used at a temperature of 25 °C and pH value of 2.3. Figure 1a,b show voltammograms obtained at 10 mV/s and 20 mV/s scan rates, within a potential window between −1.8 V and 1.0 V vs. SCE for the Ti electrode and solutions containing only indium sulfate (70 g/L In^+3^), indium sulfate and boric acid (70 g/L In^+3^, 40 g/L H_3_BO_3_), indium sulfate and sodium sulfate (70 g/L In^+3^, 30 g/L Na_2_SO_4_) and indium sulfate with both additives (70 g/L In^+3^, 40 g/L H_3_BO_3_, and 30 g/L Na_2_SO_4_). In all cases the reactions involved correspond to In^3+^ and H^+^ reduction (Equations (1) and (2)):

2H_3_O^+^ + 2e^−^ ↔ H_2_ + 2H_2_O (1)

In^+3^ + 3e^−^ ↔ In^0^ (2)

All voltammograms of Figure 1 show a single crossover point (E1) at −0.64 V that represents the equilibrium potential [52]. When the potential proceeds towards cathodic values, no HER is detected until a potential close to −1.35 V, except for case A (black line) with only indium sulfate’s presence in solution, where HER starts at about −1 V. However, after the switching point is exceeded on the reverse scanning, the deposited indium layer triggers HER at a more positive potential until the hydrogen discharge overpotential is no longer sufficient to be favored, while the reduction of indium ions occurs, preferentially producing, in general, a plateau.

**Figure 2:**
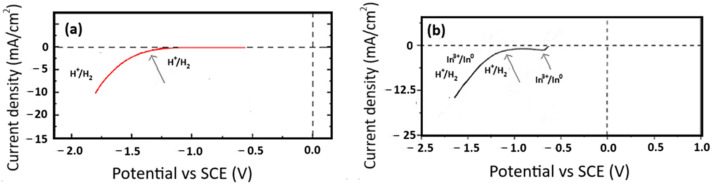
Cyclic voltammetry at 20 mV/s and room temperature using a: (**a**) blank solution containing only sulfuric acid at pH 2.3 without indium sulfate on the titanium cathode and (**b**) solution with indium sulfate at pH 2.3 on the indium cathode.

As mentioned above, when only indium is present in solution, as observed in the inset Figure 1a,b, HER triggers at less negative potential around −1.0 V, also during the forward cathodic scan. As shown in Figure 2b, it is probably due to a sudden indium reduction in a greater quantity.

Therefore, to better explain what happens in Figure 1, when indium sulfate is only in the electrolyte, two voltammograms were reported in Figure 2, considering two different metal cathodes (titanium and indium) at 25 °C and a pH of 2.3. An acidic solution without indium sulfate has been used on the titanium cathode, while on the indium cathode an acidic solution with indium sulfate has been utilized, as shown in Figure 2a,b. As expected, in the first case, it is possible to highlight that HER starts at about −1.35 V, being the unique reduction reaction. In the second case, two reactions took place, namely the indium reduction at −0.64 V with a peak at around −0.7 V, while HER starts at around −1.0 V. The comparison between Figure 2a,b could represent what happens when an indium layer is present on a titanium cathodic surface, which can act as an indium cathode. Finally, CE was calculated for all cases based on cyclic voltammetry analysis and reported in Figure 1. The results indicate that when a single additive is used, CE decreases, whereas it slightly increases when both boric acid and sodium sulfate are used, particularly at low scan rates.

Although cyclic voltammetry tests could give a CE calculation, the galvanostatic set up is quite different from cyclic voltammetry tests and the results about efficiency can be different. Thus, galvanostatic tests are necessary to assess the more realistic value of CE for the indium electrowinning process on titanium cathode cathodes. For this reason, several galvanostatic tests have been carried out to analyze the effect of both the CD and the electrolyte composition on CE and SEC.

### 3.2. Galvanostatic Tests

Galvanostatic tests performed on the titanium cathode allowed for the evaluation of the effect of different parameters on the indium deposition, such as the electrolyte composition, current density, temperature and pH. The obtained deposits were then analyzed by SEM and XRD to evaluate the morphology and crystallinity, respectively. Some evaluations have been conducted using a solution containing only indium sulfate at pH 2.3.

#### 3.2.1. Current Density Effect

**Figure 3:**
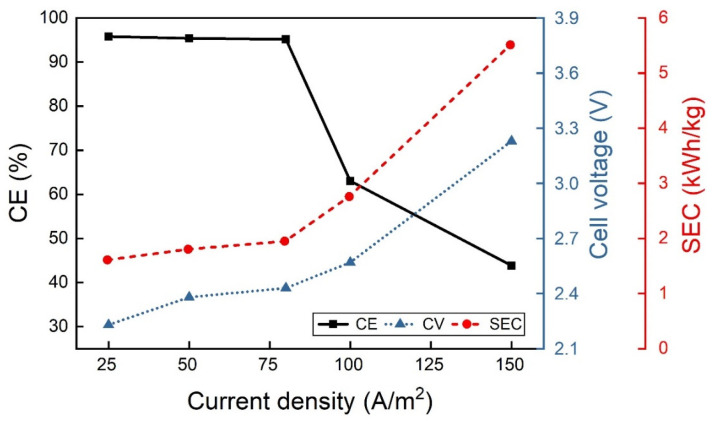
Current density effect from 25 to 150 A/m^2^ on CE, CV and SEC at 40 °C and pH 2.3 using a solution containing 70 g/L In^3+^.

A campaign of tests was performed to analyze the effect of CD by using a solution containing 70 g/L of In^3+^ at pH 2.3 and 40 °C temperature, as seen in Figure 3. The investigated CDs were 25, 50, 80, 100 and 150 A/m^2^. Increasing the CD value can change the conditions relating to the competition between the In^3+^ reduction reaction and hydrogen evolution reaction (HER). In particular, the indium electrodeposition reaction may be limited by the diffusion limit current when, especially at high CD values, the diffusive conditions for the reduction of indium at the cathode occur, determining a drastic decrease in CE. Subsequently, boric acid and sodium sulfate have been used to verify the effect of the bath composition on indium electrowinning using different CD values.

#### 3.2.2. Effect of the Electrolyte Composition

This study aims to investigate and evaluate the potential of Ti as a cathode for indium electrowinning. In order to gain deeper insights into CE (%) and SEC (kWh/kg), long-term galvanostatic tests were carried out. In the previous works [13,45,52,54,55], the additives used in the electrolyte preparation were H_3_BO_3_, Na_2_SO_4_ and Al_2_(SO_4_)_3_. Aluminum sulfate was not used in this work as preliminary tests demonstrated its ineffectiveness in indium electrowinning performed on a Ti cathode. Two main reasons allow for the selection of H_3_BO_3_ for indium electrodeposition. First, it is related to the need to work in an acidic environment, where the In^3+^ ions are stable, and the second concerns the stabilizing power of this reagent. In fact, with H_3_BO_3_ being a weak acid, it allows for better local pH control. In contrast, sodium sulfate has been widely used to improve the conductivity of solutions and the finishing surface of deposits.

**Figure 4:**
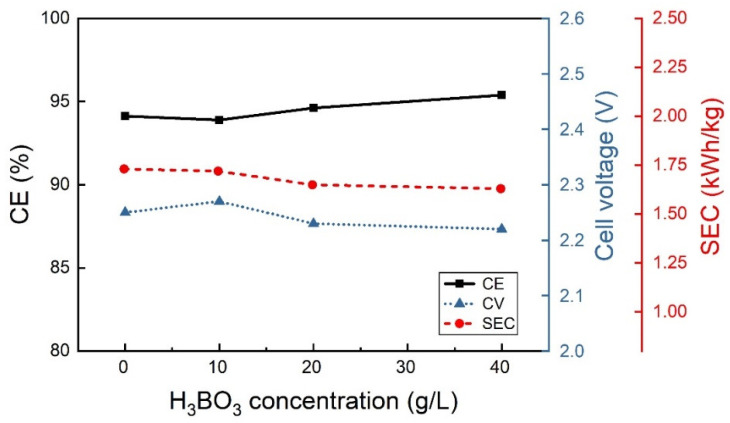
Variation of the CE, CV and SEC as boric acid concentration is changed at 25 A/m^2^, 40 °C and pH 2.3.

**Figure 5:**
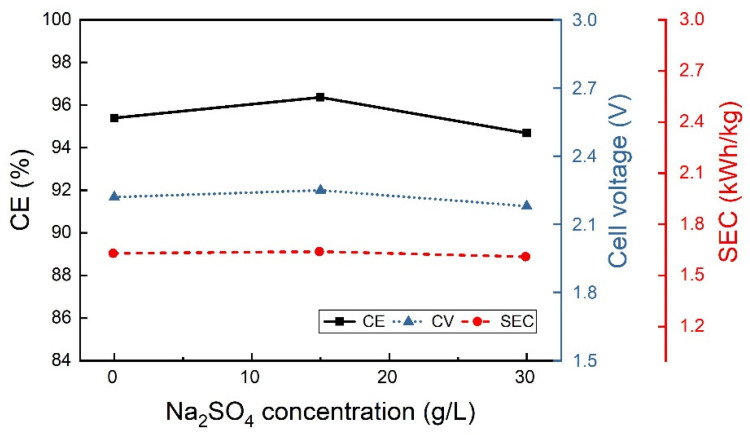
Influence of the sodium sulfate concentration on the CE, CV and SEC for the indium electrowinning using an electrolyte containing 70 g/L In^3+^ at 25 A/m^2^ at 40 °C and pH 2.3.

**Figure 6:**
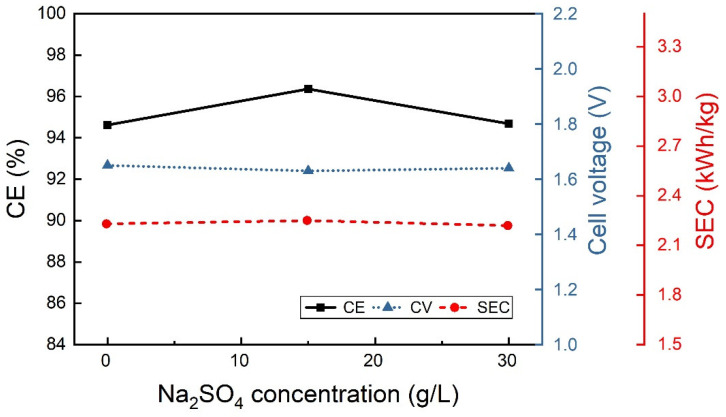
Combined effect of the sodium sulfate and boric acid on the CE, CV and SEC using a sulfate electrolyte containing 70 g/L In^3+^ and 40 g/L H_3_BO_3_ at 25 A/m^2^, 40 °C and pH 2.3.

The additives, sodium sulfate and boric acid were studied separately at the lower CD of 25 A/m^2^, 40 °C and pH 2.3. The boric acid concentration seems to have very little influence on output results (Figure 4), although the best results in terms of CE and SEC were obtained at 40 g/L of H_3_BO_3_ concentration. Under the same operating conditions, the sodium sulfate concentration also seems not to have a notable effect on CE and SEC, as shown in Figure 5. Therefore, the combined effect of both components was also studied at the same operative conditions of pH and temperature, while 40 g/L boric acid and different Na_2_SO_4_ concentrations were used (Figure 6). Accordingly, with low CD values, the additives do not seem to have significant effects.

Since CE begins to decrease drastically starting from CDs around 100 A/m^2^ (Figure 3), the impact of additives at higher CD values has been also investigated. At CD of 100 A/m^2^, CE is around 63% and decreases as CD values rise. Thus, the boric acid and sodium sulfate additives were added and the results are reported in Table 1. As shown in Table 1, increasing boric acid up to 40 g/L improves CE at higher CD values. However, adding sodium sulfate, CE drastically dropped regardless of boric acid presence. Thus, starting from an electrolytic solution with 40 g/L of boric acid, the effect of pH was analyzed using a current density equal to 100 A/m^2^.

**Figure 7:**
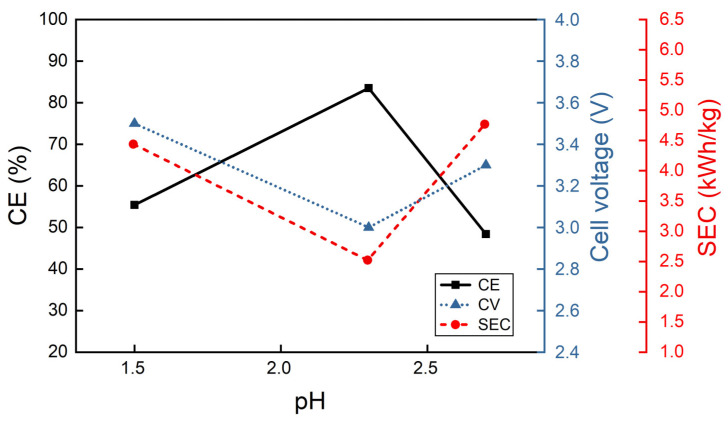
Effect of pH variation into electrolyte containing indium sulfate and boric acid at 100 A/m^2^ and 40 °C.

As can be observed from Figure 7, the current efficiency reaches its maximum at pH equal to 2.3, also confirming the results obtained in previous work using different cathodes [54]. At lower pH values, in particular, the solution is acidic favoring, at 100 A/m^2^, the hydrogen discharge reaction rather than the indium reduction. At higher pH values, the formation of insoluble indium hydroxides becomes more favored and therefore, in this case a decrease in CE also occurs.

#### 3.2.3. Effect of the Cathodic Surface Pretreatment

**Figure 8:**
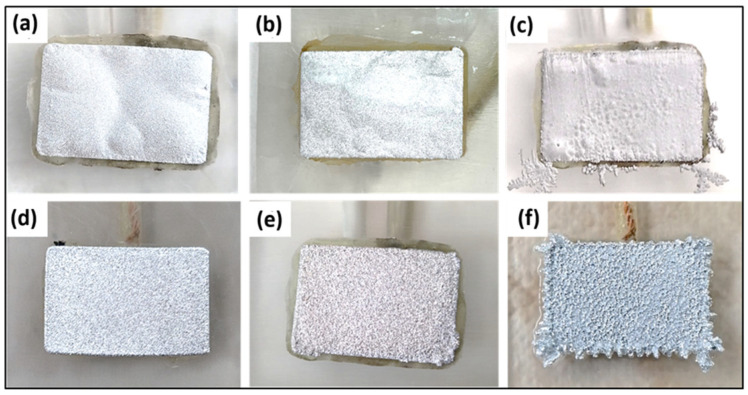
Macroscopic aspect of the indium deposits on the Ti surface, comparing samples without (**a**–**c**) and with (**d**–**f**) etching at different current densities: (**a**–**d**) 100 A/m^2^, (**b**–**e**) 150 A/m^2^ and (**c**–**f**) 200 A/m^2^.

Working, especially at high CD values, the quality of the deposit deteriorates due to its swelling and detachment from the cathode support, as observed in Figure 8. Therefore, the cathode surface was etched with 1 M hydrofluoric acid solution. This pretreatment process aimed to clean the surface and enhance the roughness, thus trying to improve the adhesion of the indium deposit to the substrate.

As seen in Table 2, which presents the results obtained in terms of CE and SEC, surface pretreatment of the cathode led to higher yields at 100 A/m^2^, even without the use of additives in the electrolyte. The resulting deposit was adherent and free of swelling. The absence of additives simplifies the process; therefore, subsequent tests were performed at 150 A/m^2^ and 200 A/m^2^ using an electrolyte containing only indium sulfate and obtaining promising results.

#### 3.2.4. Temperature Effect

The temperature effect on indium electrowinning was studied between 25 °C and 60 °C at 150 A/m^2^ and 200 A/m^2^, as seen in Table 3. Increasing temperature, a slight increase in CE at both CDs is shown, remaining an average value at around 80%. Then, it is possible to state that the temperature effect helps both indium reduction reaction and competitive HER, but it seems that for all considered CDs, even if in less evident way respect to other cathodes [13,45,54], the indium reduction is promoted more than HER. Cell voltage for both at 150 A/m^2^ and 200 A/m^2^ decreases with temperature due to a decrease of ohmic, diffusion and activation overpotentials of electrolytical bath and consequently also SEC values for the indium electrowinning diminishes as temperature rises. To the best of our knowledge, the temperature promotes an improvement in the kinetic response for the electrodeposition reaction near the cathodic surface; however, increasing temperatures above 40 °C can adversely affect the process due to the formation of undesirable mist around the electrolytic cell. From Table 3, the most significant drop of the CV as temperature increases is observed with those values at high CDs, passing from about 4.0 to 3.5 V at 25 °C and 60 °C, respectively. Furthermore, a similar trend is seen in SEC values; as already said, they decline as the temperature increases.

### 3.3. SEM Evaluation of Indium Deposits

In the following analysis some tests having performed good results with higher CE values have been taken into account, while tests with lower CE have been considered only for a necessary comparison.

**Figure 9:**
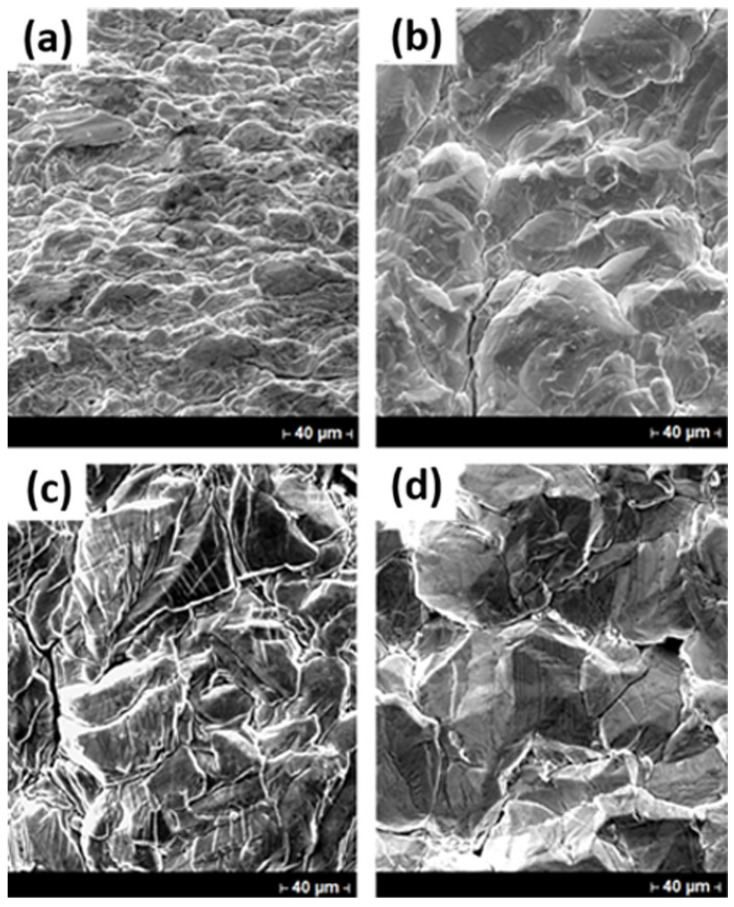
SEM micrographs of indium deposits obtained using: (**a**,**b**) 70 g/L In^3+^ and (**c**,**d**) 70 g/L In^3+^, 30 g/L Na_2_SO_4_ at pH 2.3 and 40 °C for 22 h. Deposits on the left column of figure corresponds to tests performed at 25 A/m^2^ CD, while the right one corresponds to tests performed at 100 A/m^2^.

In Figure 9, it is possible to observe SEM micrographs for indium deposits obtained without boric acid at 25 A/m^2^ and 100 A/m^2^ remaining constant the pH of 2.3, concentration of 70 g/L In^3+^, temperature of 40 °C for 22-h of electrowinning process. Without boric acid, the morphology of deposits is planar at 25 A/m^2^ (Figure 9a); as the CD increases the grains become more rounded (100 A/m^2^, Figure 9b). It should be emphasized that the grains are bigger at higher CDs, but in both cases deposits are compact. On the other hand, the addition of Na_2_SO_4_ exhibits a change in the surface appearance of deposits at different CD values, as shown in Figure 9c,d. The addition of Na_2_SO_4_ showed deposits with more compact with a like-lamellar structure as CD increases (Figure 9d).

**Figure 10:**
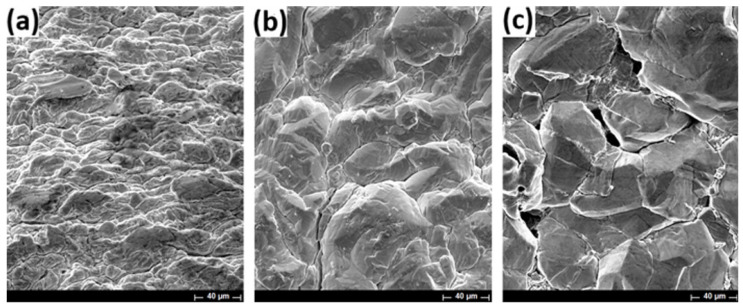
Micrographs of indium deposits obtained using 70 g/L In^3+^, pH 2.3 at 40 °C for 22 h. SEM micrographs are presented varying the CD at (**a**) 25 A/m^2^, (**b**) 100 A/m^2^, (**c**) 150 A/m^2^ with boric acid.

In the case of the boric acid addition to the electrolytic solution (Figure 10a–c), a similar morphology is obtained at low and high CDs. In particular, the presence of boric acid enhances the microstructure by producing highly defined surface features.

**Figure 11:**
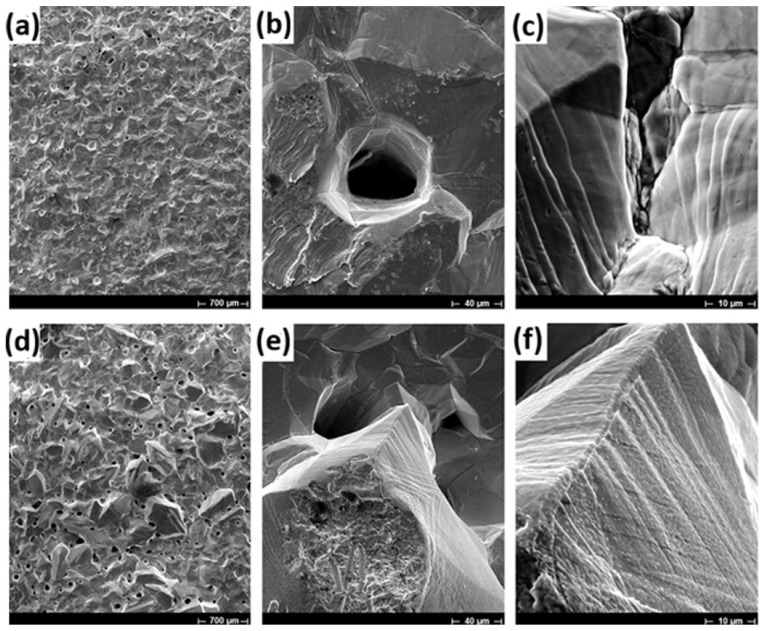
SEM micrographs of indium deposits using 70 g/L In^3+^, pH of 2.3, 40 °C, an etched Ti cathode by 1M HF and deposition time of 22 h at different CD (**a**–**c**) 100 A/m^2^ and (**d**–**f**) 200 A/m^2^.

The indium deposit obtained on the etched Ti cathode with 1M HF at 100 A/m^2^ (Figure 11a–c) and (Figure 11d–f). Deposits elaborated at high CDs present a very homogeneous and compact morphology with homogeneous lamellar microstructure at a higher magnification (Figure 11c). Similar behavior occurs at 200 A/m^2^. In addition, working at high CDs it is possible to observe the formation of rounded holes related to intense hydrogen evolution (Figure 11a,b,d,e).

**Figure 12:**
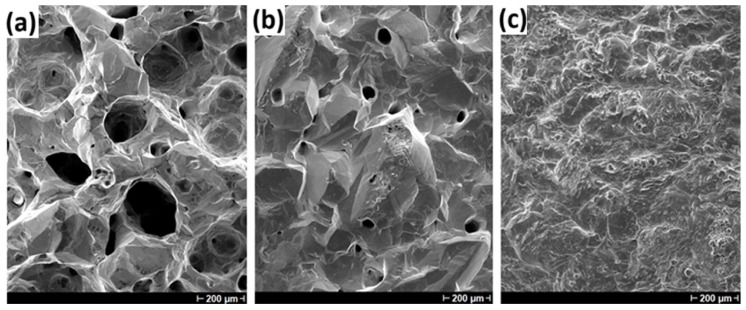
Morphology of indium deposits obtained by using an etched Ti cathode, at 200 A/m^2^, 70 g/L In^3+^ and pH 2.3 for 22 h. The considered temperatures were (**a**) 25, (**b**) 40 and (**c**) 60 °C.

Figure 12 shows the morphology of indium deposits obtained at different temperatures (25, 40 and 60 °C) using a CD of 200 A/m^2^. An intense hydrogen evolution at room temperature is observed due to a morphology full of holes (Figure 12a) showing cavities with diameters higher than 200 μm, while the temperature increase maintains a more compact microstructure (Figure 12c). The HER is present during all the experimental campaigns due to high CDs, but the temperature increase allows bubbles to move, collapse or detach faster. Since the temperature can modify resistive phenomena in the bath, it can alter the mobility for both indium and hydrogen ions or even accelerate the collapse of bubbles. On the whole, as corroborated in Table 3, increasing the temperature makes it possible to increase CE and lower SEC values, but its contribution is definitely positive also for the indium deposit morphology and aspect.

### 3.4. XRD Analysis

**Figure 13:**
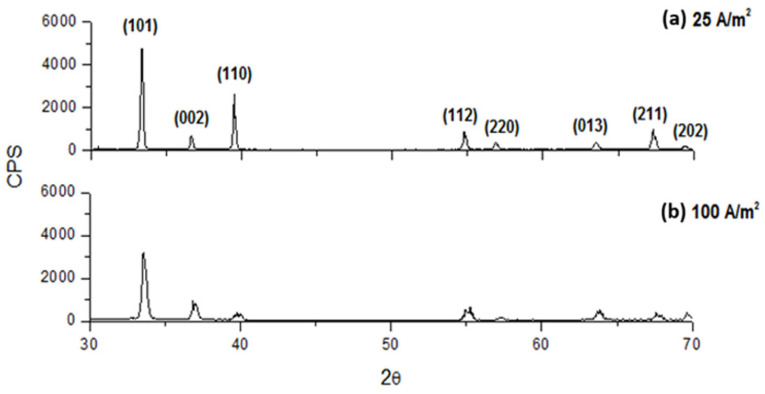
Diffractograms for indium deposits obtained at pH 2.3, 40 °C with an electrolyte containing only 70 g/L In^3+^ and different CDs: (**a**) 25 A/m^2^ and (**b**) 100 A/m^2^.

**Figure 14:**
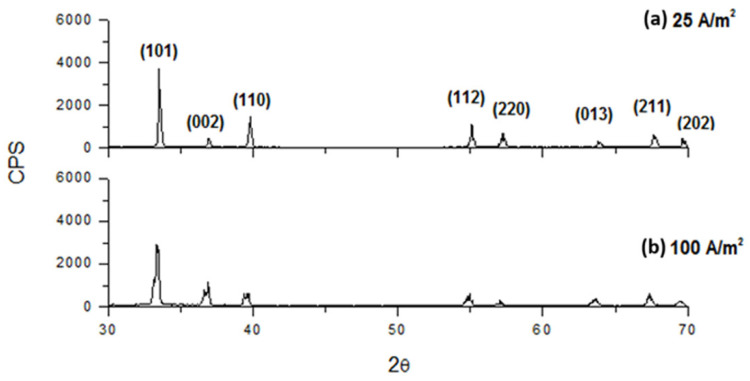
Diffractograms for indium deposits obtained at pH 2.3, 40 °C with an electrolyte containing 70 g/L In^3+^ and 30 g/L Na_2_SO_4_ and different CDs: (**a**) 25 and (**b**) 100 A/m^2^.

**Figure 15:**
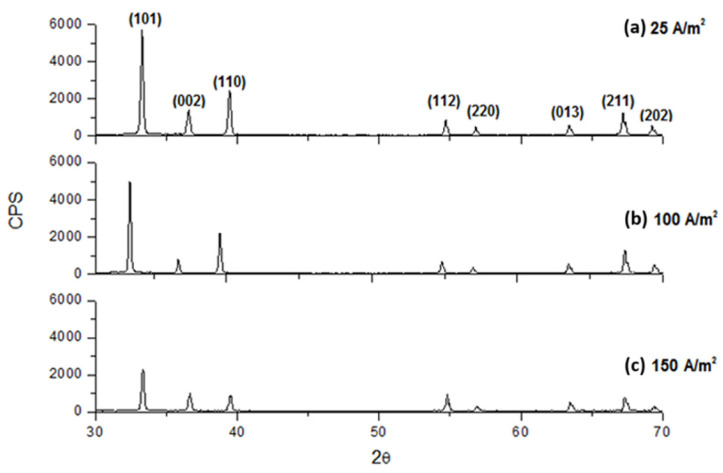
Diffractograms for indium deposits obtained at pH 2.3, 40 °C with an electrolyte containing 70 g/L In^3+^ and 40 g/L H_3_BO_3_ and different CDs: (**a**) 25, (**b**) 100 A/m^2^ and (**c**) 150 A/m^2^.

The crystallographic characteristics of the deposits at different CDs showed slight changes varying in the electrolyte composition. As the CD increased, the crystallinity generally decreased. Indeed, as it results from Figure 13, Figure 14 and Figure 15 for an electrolyte containing only 70 g/L In^3+^ or 70 g/L In^3+^ and 30 g/L Na_2_SO_4_ or 70 g/L In^3+^ and 40 g/L H_3_BO_3_, the intensity of diffractograms decreases progressively as CD increases. Moreover, the diffractograms, obtained at the highest current densities, show a slight change in the succession of the peak heights, independently from all other operative conditions. In particular, this analysis shows that the main preferential orientation is (101) and it is also the most intense peak for each CD, while the relative intensities of the second and third peaks change, with peak (110) tending to be more intense than peak (002) at low current densities, while at higher current densities the situation is reversed. Lastly, the sequence of intensities of the last five peaks is almost always unchanged when the composition and current density change.

From Figure 14, it appears that working at 25 A/m^2^ or 100 A/m^2^, the addition of 30 g/L of sodium sulfate seems to have only slightly influence on the peaks intensity and the deposit crystallinity is practically unchanged.

**Figure 16:**
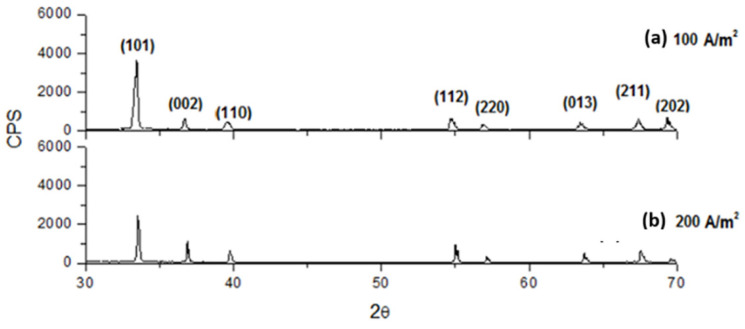
Diffractograms for indium deposits obtained at pH 2.3, 40 °C with an electrolyte containing 70 g/L In^3+^ using the etched Ti cathode at (**a**) 100 A/m^2^ and (**b**) 200 A/m^2^.

**Figure 17:**
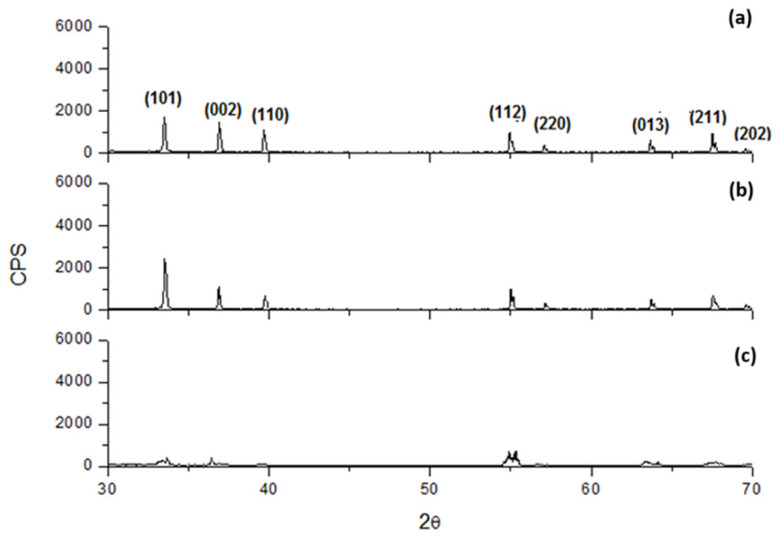
Diffractograms of indium electrowinning performed at different temperatures (**a**) 25 °C, (**b**) 40 °C and (**c**) 60 °C using the etched Ti cathode at 200 A/m^2^ and a sulfate electrolyte containing 70 g/L In^3+^ at pH 2.3 for 22 h.

In contrast, the addition of 40 g/L of boric acid causes a little increase in crystallinity, both at 25 A/m^2^ and 100 A/m^2^, as shown in Figure 15 by the presence of more intense peaks. Moreover, as it results in Figure 16, by first etching the titanium cathode with HF without adding any additives in solution, the aspect of the obtained diffractogram at 100 A/m^2^ seems to be very similar to those in which etching is absent, highlighting a comparable crystallinity. In general, therefore, it can be said that working with higher CDs results in less crystalline deposits. Boric acid slightly tends to increase crystallinity, while sodium sulfate has no effect on it. Finally, even if, as results from Figure 12, the deposit obtained at higher temperature and 200 A/m^2^ seems to be more compact with respect to those obtained at a lower temperature, it seems, on the other hand, also amorphous, as shown in Figure 17.

Accordingly with the shown diffractograms, it is possible to state that, as reported in the literature [57,58,59], a change of peak intensities, due to a change in preferential crystal growth, as well as a decrease in peaks height, related to a minor crystallinity, can be attributed to different phenomena: at higher current density, as well as at higher temperature the nucleation rate increases producing at the same time a higher number of nuclei, which in turn generate a larger number of finer crystallites increasing, on one hand, the stress inside the deposit and, on the other hand, decreasing the internal crystalline order. Moreover, at a higher current density or higher temperature HER is enhancing, increasing the interaction between the deposited metal ion and absorbed H atoms and favoring, in this way, some different preferential crystal growth. These phenomena together can produce the differences between XRD results.

Considering all the results shown above, it is possible to affirm that in comparison with other cathodic materials, titanium presents significant advantages for the indium electrowinning [13,45,52,54]. Productivity is one of the most positively affected outcomes, given that titanium, used as a cathode, can operate at higher current densities with respect to other metals such as stainless steel and nickel. Titanium enables the formation of uniform deposits, achieving acceptable values of high CE and low SEC [13,45,54,55]. Furthermore, the surface features of titanium allow the indium electrowinning to be carried out without the need for the additives in the electrolytic bath, even after performing a surface etching. This could simplify the industrial operation and reduce associated costs. Therefore, considering similar operating conditions and high current densities for the indium electrowinning, the titanium cathode demonstrates higher productivity, offering a clear economical advantage over other cathodic materials.

## 4. Conclusions

Experimental tests performed on the indium electrowinning from sulfate solutions, using a titanium cathode, allowed obtaining a high productivity while maintaining high CE and low SEC values. In particular, the best performances with Ti cathode cathodes were achieved at 100 A/m^2^, 40 °C and pH 2.3 by using a sulfate electrolyte containing 70 g/L In^3+^, 40 g/L H_3_BO_3_ with CE of around 84% and SEC of 2.5 kWh/kg. Furthermore, higher current densities up to 200 A/m^2^ can be utilized by etching the titanium cathodic surface with 1 M HF and working at 40 °C and pH 2.3. In these conditions and without adding additives, significant results such as values of CE and SEC around 80% and 2.8 kWh/kg, respectively, can be achieved. The morphology of the deposits seems not to be influenced by the presence of additives, while an increase in CD leads to an increase in grain size, maintaining the same lamellar growth. The appearance of the samples obtained at the highest CD is positively affected by the temperature rise, which decreases the presence of pores produced by HER. Finally, working with different electrolyte compositions, XRDs performed on the resulting samples showed only minimal variations in peak intensities. In particular, increasing the CD results in a decrease in peak intensities, which, however, maintain the same preferential orientation (101). Lastly, at the highest CD and temperature, the diffractogram shows an almost amorphous structure.

## Figures and Tables

**Table 1:** Comparative effect of boric acid and sodium sulfate on the indium electrowinning at high CDs, 40 °C and pH 2.3.

H_3_BO_3_ (g/L)	Na_2_SO_4_ (g/L)	CD (A/m^2^)	CE (%)	SEC (kWh/kg)
0	0	100	59.0	3.5
5	0	100	63.0	2.7
40	0	100	83.5	2.5
0	0	150	43.8	5.5
40	0	150	63.5	3.7
0	30	100	33.1	6.5
40	30	100	23.7	9.1

**Table 2:** Comparing CV, CE and SEC for between 100 A/m^2^ and 200 A/m^2^ at 40 °C and pH 2.3 with and without etching. The etching was performed by 1 M HF for 30 s.

Pretreatment	CD (A/m^2^)	CV (V)	CE (%)	SEC (kWh/kg)
-	100	2.4	63.0	2.7
Etching	100	3.1	77.6	2.8
-	150	3.5	43.8	5.5
Etching	150	3.2	79.3	2.8
-	200	3.4	38.1	6.2
Etching	200	3.5	71.8	3.4

**Table 3:** Evaluation of temperature on the indium electrowinning using a Ti cathode etched by 1 M HF using an electrolyte containing 70 In^3+^ g/L at 150 A/m^2^ and 200 A/m^2^.

Temperature (°C)	Current Density (A/m^2^)	CV (V)	SEC (kWh/kg)	CE (%)
25	150	3.5	3.3	77.3
25	200	4.0	3.8	74.2
40	150	3.6	2.8	79.3
40	200	3.7	3.6	71.3
60	150	3.4	2.8	85.0
60	200	3.5	3.4	71.8

## Data Availability

The original contributions presented in this study are included in the article. Further inquiries can be directed to the corresponding author.
